# The Effect of Dietary Supplementation with Plant Sterols on Total and LDL-Cholesterol in Plasma Is Affected by Adherence to Mediterranean Diet: Insights from the DESCO Randomized Clinical Study

**DOI:** 10.3390/nu15214555

**Published:** 2023-10-27

**Authors:** Arrigo F. G. Cicero, Federica Fogacci, Marina Giovannini, Elisabetta Rizzoli, Elisa Grandi, Sergio D’Addato, Claudio Borghi

**Affiliations:** 1 Hypertension and Cardiovascular Risk Research Group, Medical and Surgical Sciences Department, University of Bologna, 40138 Bologna, Italy; arrigo.cicero@unibo.it (A.F.G.C.); marina.giovannini3@unibo.it (M.G.); elisabetta.rizzoli@unibo.it (E.R.); elisa.grandi@unibo.it (E.G.); sergio.daddato@unibo.it (S.D.); claudio.borghi@unibo.it (C.B.); 2 IRCCS Azienda Ospedaliero, Universitaria di Bologna, 40138 Bologna, Italy; 3 Italian Nutraceutical Society (SINut), 40100 Bologna, Italy

**Keywords:** dietary supplementations, phytosterols, plant sterols, cholesterol, apolipoproteins, hypercholesterolemia, mediterranean diet, clinical trial

## Abstract

Plant sterols are well-known natural lipid-lowering agents. The DESCO (Diet and plant sterols in the control of cholesterolemia) study was a single-center, randomized, double-blind, placebo-controlled, two-way crossover clinical trial designed to investigate the effect of a once-a-day ready-to-drink dietary supplement containing 2.5 g of phytosterols on the lipid profile, also in relation to the quality of the diet, in a cohort of 50 Italian individuals with polygenic hypercholesterolemia and low global cardiovascular risk. Eligible individuals were enrolled in a run-in period of 2 weeks. Then, participants who qualified for continuation in the study were randomly allocated (1:1) to a 3-week treatment with either phytosterols or placebo. After a 2-week washout period, enrolled individuals were crossed over to receive the alternative treatment. Dietary supplementation with phytosterols was associated with significant improvement in plasma levels of total cholesterol (TC; −11.8 ± 4.0 mg/dL, *p* = 0.016), low-density lipoprotein cholesterol (LDL-C; −7.8 ± 7.7 mg/dL, *p* = 0.021), and apolipoprotein B-100 (Apo B-100, −3.7 ± 4.1 mg/dL, *p* = 0.048) compared to baseline. The changes in TC and LDL-C were also significant compared to placebo, and greater adherence to the Mediterranean diet was significantly associated with greater reductions in LDL-C. Dietary supplementation with phytosterols was well tolerated and adherence to treatment was high. According to the findings of DESCO, the once-a-day ready-to-drink dietary supplement we tested is able to quickly and significantly decrease plasma levels of TC, LDL-C, and Apo B-100, with a greater effect in individuals more adhering to the Mediterranean dietary pattern.

## 1. Introduction

Atherosclerotic cardiovascular diseases (ASCVDs) still represent a leading cause of death and morbidity in most European and North American countries, and elsewhere in the world [1]. A large body of evidence suggests that unhealthy lifestyles and hypercholesterolemia are among the main risk factors for ASCVD [2]. In this context, the improvement in dietary habits and the optimization of the levels of low-density lipoprotein cholesterol (LDL-C) in the blood seem to be effective tools to reduce ASCVD risk in the general population [3]. In particular, the Mediterranean diet—consisting of a high quantity of fish, unsaturated fats, whole grains, fruits and vegetables, nuts, and legumes—has consistently been shown to be associated with a reduced CV morbidity and mortality and potential surrogate markers of ASCVD in meta-analyses, cohort studies and randomized clinical trials [4,5]. Of course, pharmacological treatment should always be considered for the management and treatment of hypercholesterolemia, but its cost-effectiveness and risk–efficacy ratios are more favorable in patients at high CV risk rather than in those at low risk [6]. In mildly hypercholesterolemic patients not requiring statin treatment, a healthy lifestyle could be then supported by the supplementation with some dietary bioactive compounds of proven efficacy. In this respect, a recent meta-analysis of 13 randomized clinical trials concluded that the supplementation of plant sterols is safe and can reduce LDL-C by an average of 12.14 mg/dL (95% confidence interval (CI): 8.98, 15.29 mg/dL) [7], with greater improvements for doses of 2 gr/day and in individuals with LDL-C plasma levels >140 mg/dL at baseline [7]. Based on the available data, the European Commission Regulation (EU) approved the health claim proposed by the European Food Safety Authority (EFSA) regarding the cholesterol-lowering effect of products providing 1.5–3 g/day of plant sterols or stanols [8].

The latest International guidelines of the European Society of Cardiology (ESC) and the European Society of Atherosclerosis (EAS) recommend the daily consumption of functional food with 2 g of plant sterols/stanols (i) in individuals at low or intermediate CV risk and high cholesterol levels who do not qualify for pharmacotherapy according to their global CV risk; (ii) in addition to pharmacotherapy in high and very high risk patients who failed to achieve LDL-C-goals on statins or who cannot be treated with statins; and (iii) in individuals with familial hypercholesterolemia (FH) [9,10]. Interestingly, insights from the Hamburg City Health Study (*n* = 7223) highlight a trend toward the use of phytosterol-enriched products by individuals at increased CV risk independent of income [11]. However, a dose–effect relationship of plant stanols in higher doses than currently recommended by the international guidelines has been demonstrated by several clinical studies and a comprehensive meta-analysis [12]. Moreover, a recent population-based study conducted in Poland (*n* = 5690) shows that habitual dietary intake of plant sterols may be associated with a lower chance of developing ASCVD, particularly in men [13].

It has to be acknowledged that most of the available evidence on this issue comes from clinical trials conducted in North America and Northern and Central Europe, where the consumption of meat and dairy products is high and the diet tends to be low in whole grains, fruit, and vegetables [12]. However, the add-on effect of dietary supplementation with phytosterols in individuals already following a plant-based diet (e.g., the Mediterranean diet) has not yet been fully investigated, even though this is the most suggested dietary pattern to reduce CV risk. Moreover, previously published clinical studies mainly tested functional foods enriched in phytosterols (e.g., phytosterol-enriched milk and dairy spreads) that had to be taken more times a day and needed to be stored and consumed following specific instructions to avoid losing their quality and nutritional value [14]. To fill the gaps in the available knowledge, we carried out a double-blind, placebo-controlled, randomized clinical trial testing the short-term lipid-lowering effect of a new once-a-day ready-to-drink dietary supplement containing 2.5 g of plant sterols in Italian individuals with mild-to-moderate hypercholesterolemia. We also investigated whether the LDL-lowering effect of the tested dietary supplement was affected by the degree of adherence of the study’s participants to the Mediterranean diet.

## 2. Materials and Methods

### 2.1. Study Design and Participants

DESCO (Diet and plant sterols in the control of cholesterolemia) is a randomized, double-blind, placebo-controlled, two-way crossover clinical study that involved a sample of Italian free-living individuals with polygenic hypercholesterolemia, who were recruited from the Lipid Clinic of the S. Orsola Malpighi University Hospital, Bologna, Italy.

Participants were required to be aged between 20 and 65 years, with moderately high levels of plasma cholesterol (total cholesterol (TC) > 200 mg/dL and <240 mg/dL and/or LDL-C > 130 mg/dL and <160 mg/dL) and an estimated 10-year cardiovascular risk <5% according to the SCORE (Systematic COronary Risk Evaluation) risk charts for countries at moderate risk [15], not requiring lipid-lowering treatments. Individuals with high to very high CV risk [15] and patients with uncontrolled hypertension (i.e., systolic blood pressure (SBP) and/or diastolic blood pressure (DBP) > 190/100 mmHg) or affected by diseases potentially affecting lipid metabolism [16] were excluded from the study. Further exclusion criteria included triglycerides (TG) > 200 mg/dL, obesity (body mass index (BMI) > 30 Kg/m^2^), diabetes mellitus, chronic kidney disease (defined as an estimated glomerular filtration rate (eGFR) < 60 mL/min), a positive test for human immunodeficiency virus (HIV) or hepatitis B/C/E, uncontrolled thyroid diseases, history of malignancies, use of medications or nutritional supplements that altered plasma lipids (e.g., statins, ezetimibe, omega-3 fatty acids, fibrates and bile acid resins), alcoholism, pregnancy, and breastfeeding.

Enrolled subjects were asked to follow their habitual diet for 2 weeks before randomization and during the study, and to avoid consumption of other supplements or functional foods containing substances with effects on cholesterol (red yeast rice, berberine, beta-glucan, etc.). At baseline and on the day of crossover, the study participants received adequate doses of either phytosterols or placebo to complete one of two 3-week treatment sequences. The crossover to the second treatment was preceded by a 2-week washout period.

Before and after each treatment sequence, participants were evaluated for clinical status, and via the execution of a physical examination and laboratory and hemodynamic analyses.

**Figure 1:**
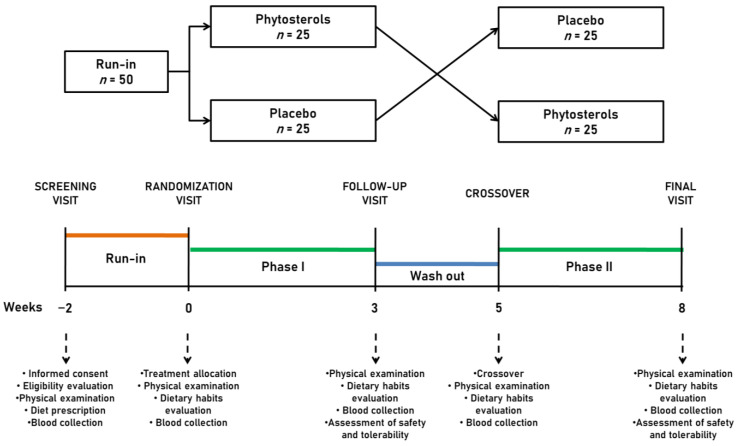
Study timeline.

The study timeline is described in detail in Figure 1.

The study fully complied with the ethical guidelines of the Declaration of Helsinki and with The International Council for Harmonization of Technical Requirements for Registration of Pharmaceuticals for Human Use (ICH) Harmonized Tripartite Guideline for Good Clinical Practice (GCP). The study protocol was approved by the Ethical Committee of the University of Bologna and registered in ClinicalTrials.gov (ID: NCT05265455). All participants signed a written informed consent to participate.

### 2.2. Treatment

After a 2-week period of diet standardization, enrolled volunteers were randomized to receive either a daily 15 mL stick of a ready-to-drink formulation containing 2.5 g of phytosterols and 0.33 mg of thiamin in demineralized water and sodium carboxymethylcellulose as thickener (1 stick), or an indistinguishable placebo (Table 1).

The study products were manufactured and packaged in accordance with Quality Management System ISO 9001:2008 and the European Good Manufacturing Practices (GMP), satisfying requirements in “Code Of Federal Regulation” title 21, volume 2, part 111.

Randomization was performed centrally using computer-generated codes. Participants and investigators were blinded to the group assignment. Randomization codes were kept in a sealed envelope that was opened after study completion and data analysis.

For each treatment sequence, each patient was provided with boxes containing 30 sticks and was instructed to take a stick once daily, just before the main meal. At the end of each treatment sequence, all unused sticks were retrieved for inventory. Participants’ compliance was assessed by counting the number of returned sticks and calculating the ratio between the number of days from the first day of treatment and the number of sticks consumed by the participants.

### 2.3. Assessments

#### 2.3.1. Clinical Data and Anthropometric Measurements

Information gathered in the participants’ history included presence of ASCVD and other systemic diseases, allergies, and medications. Validated semi-quantitative questionnaires were used to assess demographic variables, recreational physical activity, and smoking habits [17]. Participants’ adherence to the Mediterranean diet was assessed through a self-administered 15-item questionnaire (QueMD) that was previously validated in the Italian population [18]. Following the method proposed by Gnagnarella et al. [18], we used data from the QueMD to calculate the aMED (alternate Mediterranean) score, assigning 1 point to participants reporting consumptions above the average Italian National levels [19] for intakes of each of the following foods: vegetables, fresh fruits, dried fruits, wholegrain cereals, pulses, fish, and olive oil. We also assigned 1 point to those consuming red and processed meat ≤1–3 times/week and 1 point for men drinking 1–2 glasses of wine per day or women drinking a limited amount of wine (>0 < 1/day). The calculated aMED score ranged from 0 (minimal adherence to the Mediterranean diet) to 9 (maximal adherence). Participants were then classified as having a low or high adherence to the Mediterranean diet based on the median sample aMed score (5.8). Data were handled in compliance with the company procedure IOA87.

Waist circumference (WC) was measured in a horizontal plane at the end of a normal expiration, at the midpoint between the inferior margin of the last rib and the superior iliac crest. Height and weight were respectively measured to the nearest 0.1 cm and 0.1 Kg, with subjects standing erect, eyes directed straight, and wearing light clothes and bare feet. BMI was calculated as body weight in kilograms, divided by height squared in meters (Kg/m^2^). Finally, the index of central obesity (ICO) was calculated as the ratio of WC and height [20].

#### 2.3.2. Laboratory Analyses

Biochemical analyses were carried out on venous blood withdrawn after overnight fasting (at least 12 h). Plasma was obtained by addition of disodium ethylenediaminetetraacetate (Na_2_EDTA) (1 mg/mL) and blood centrifugation at 3000 RPM for 15 min at 25 °C.

Immediately after centrifugation, trained personnel performed laboratory analyses according to standardized methods [21]. The following parameters were directly assessed: TC, TG, high-density lipoprotein cholesterol (HDL-C), and apolipoprotein B-100 (Apo B-100).

LDL-C was obtained using the Friedewald formula. Non-HDL cholesterol (Non-HDL-C) resulted from the difference between TC and HDL-C. Lipid accumulation product (LAP) was calculated as (WC − 65) × TG (expressed in mmol/L) for men and (WC − 58) × TG (expressed in mmol/L) for women [22]. Finally, the visceral adiposity index (VAI) was calculated as follows [23]: (WC (cm)/(39.68 + (1.88 × BMI))) × (TG (mmol/L)/1.03) × (1.31/HDL-C (mmol/L)) for males; and (WC/(36.58 + (1.89 × BMI))) × (TG/0.81) × (1.52/HDL-C) for females.

#### 2.3.3. Blood Pressure Measurements

Blood pressure (BP) was measured in accordance with the recommendations of the International Guidelines for the management of arterial hypertension [24]. Resting systolic and diastolic BP were measured with a validated oscillometric device, and a cuff of the appropriate size applied on the right upper arm. To improve detection accuracy, three BP readings were sequentially obtained at 1 min intervals. The first reading was discarded, and the average between the second and the third reading was recorded as the study variable.

#### 2.3.4. Assessment of Safety and Tolerability

Safety and tolerability were evaluated via a continuous monitoring during the study, to detect any adverse event, clinical safety, laboratory findings, vital sign measurements, and physical examinations. A blinded, independent expert clinical event committee was appointed by the principal investigator to categorize the adverse events that could possibly be experienced during the trial as not related, unlikely related, possibly related, probably related, or definitely related to the tested treatment [25].

### 2.4. Statistical Analysis

Data were analyzed using intention to treat by means of the Statistical Package for Social Science (SPSS) 25.0, version for Windows.

The sample size was calculated for the change in LDL-C. By enrolling 45 subjects in this two-treatment crossover study, the probability is 90 percent that the study would detect a treatment difference at a two-sided 0.5 significance level, if the true difference between treatments is 5%. This is based on the assumption that the within-patient standard deviation of the response variable is 18. A total sample size of 50 subjects (25 subjects/treatment sequence) was then used in the study to allow for a dropout rate of 10%.

The Kolmogorov–Smirnov test was used to test the normality distribution of the studied variables. Baseline characteristics of the population were compared using Levene’s test followed by the independent Student’s *t* test and by the χ^2^ test followed by Fisher’s exact test. Between-group differences were assessed using repeated-measures ANOVA (analysis of variance) followed by Tukey’s post hoc test. All data are expressed as means ± standard deviations. A multiple linear regression model was also used to identify the predictors of treatment response (i.e., LDL-C reduction) to dietary supplementation with phytosterols. All tests were two-sided. A *p* level of <0.05 was considered significant for all tests.

## 3. Results

### 3.1. Efficacy Analysis

**Figure 2:**
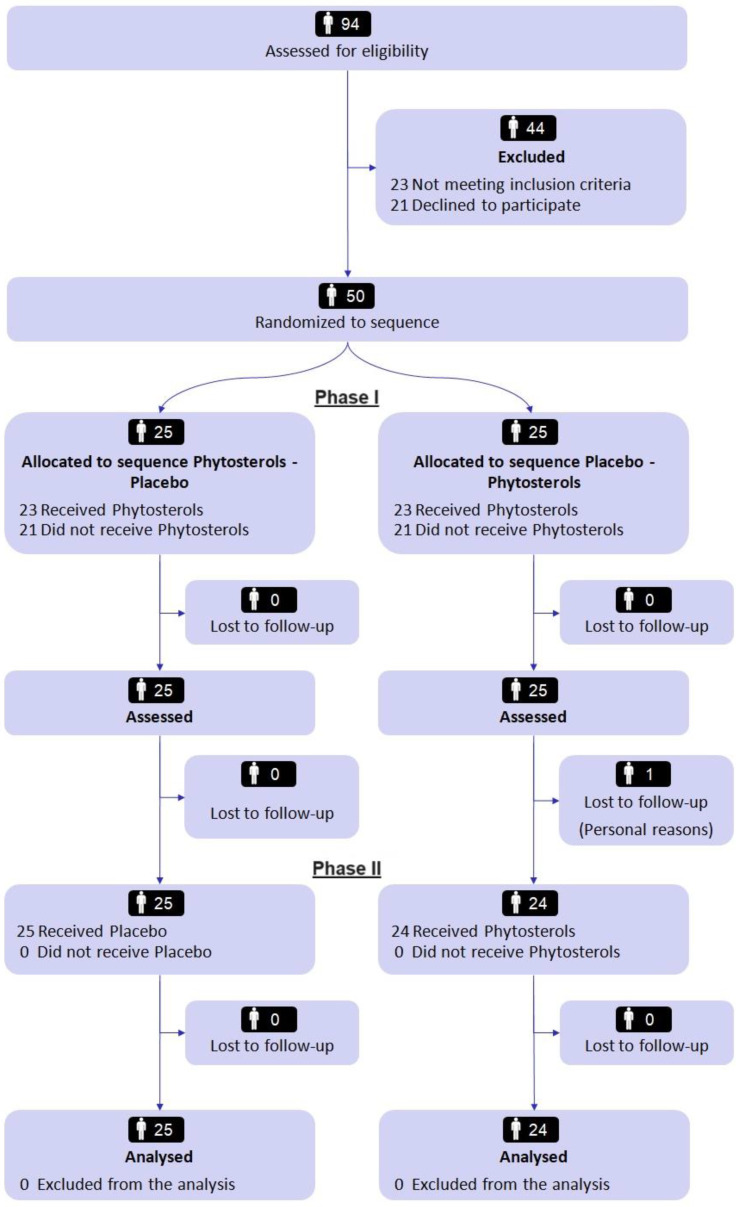
CONSORT (Consolidated Standards of Reporting Trials) flow diagram modified for crossover clinical studies.

A total of 94 volunteers was screened, and 50 subjects underwent randomization from October 2021 through April 2022. Forty-nine enrolled subjects successfully completed the trial according to the study design (Figure 2).

The mean compliance with the treatment was 94 ± 2% in the active treated group and 90 ± 3% in the placebo group.

The final distribution by sex did not show any significant differences between groups (*p* > 0.05), with 35 women allocated to placebo and 34 women allocated to the active treatment during the different study phases and no detectable interaction effect.

The pre-run-in and post-run-in characteristics of the enrolled subjects are summarized in Table 2. During the run-in phase, non-significant changes in anthropometric and laboratory data were registered (*p* always >0.05).

The study groups were adequately matched for anthropometric, hemodynamic, and laboratory parameters at baseline (Table 3). Overall, no statistically significant changes were recorded in the dietary habits of the enrolled individuals from randomization until the end of the study, according to the aMED score (Table 3).

At the end of the trial, no significant change was recorded after both treatments as regards anthropometric and hemodynamic variables (Table 3).

No significant change was observed during the placebo-controlled phase (Table 4).

Dietary supplementation with phytosterols was associated with significant improvement in plasma levels of TC, LDL-C, and Apo B-100 compared to baseline. The changes in TC (Cohen’s d = 0.04) and LDL-C (Cohen’s d = 0.04) were also significant compared to placebo (Table 4). Greater adherence to the Mediterranean diet significantly correlated with greater reductions in LDL-C in the study cohort. In particular, individuals with low aMED score (<5.8 points) experienced, on average, a smaller reduction in both TC (−4.5 ± 5.3%) and LDL-C (−6.3 ± 9.1%) than individuals with high aMED score (≥5.8 points; TC −12.3 ± 8.6% and LDL-C −9.2 ± 11.8%). As significant predictors of change in LDL-C, the multiple regression model identified dietary supplementation with phytosterols, baseline LDL-C levels, and aMED score (*p* < 0.05) (Table 5).

Sex, BMI, and Apo B-100 were not associated with the effect size.

### 3.2. Safety Analysis

All participants completed the clinical trial according to the study design, except one patient, who was lost to follow-up due to personal reasons. No treatment-emergent adverse event was reported nor laboratory abnormalities occurred during the study.

## 4. Discussion

During the last two decades, a large and compelling body of evidence has accumulated in support of the use of lipid-lowering dietary supplements in patients at low ASCVD risk, for whom pharmacological treatment is not recommended [26,27]. Among nutraceuticals and functional foods with a recognized LDL-C-lowering effect, plant sterols are the most used due to their good tolerability profile and the negligible risk of pharmacological interactions, either alone or when co-administered with other lipid-lowering drugs, including statins [28,29].

Plant sterols impair cholesterol absorption from the bowel via different mechanisms: by competing with dietary and biliary cholesterol for micellar solubilization in the intestinal lumen, impairing intestinal cholesterol absorption, altering the conversion of bile acids into secondary bile acids, and lowering the bile acid hydrophobic/hydrophilic ratio [30]. In addition to its well-known LDL-C-lowering effect, dietary supplementation with phytosterols has also been associated with significant improvements in pro-atherogenic and anti-atherogenic apolipoproteins. In this regard, a large meta-analysis of 37 randomized clinical trials with 51 arms has recently shown that phytosterols increase plasma levels of Apo A-I and Apo C-II, and decrease Apo B-100, the Apo B-100/Apo A-I ratio, and Apo E, without affecting plasma levels of Apo A-II and Apo C-III [31]. Moreover, dietary supplementation with plant stanol esters has been found to significantly reduce both the LDL aggregation susceptibility and the degree of LDL binding to proteoglycans, which in turn results in a reduced risk of ASCVD [32]. Phytosterols also reduce plasma concentration of endothelin-1 by 11% [33]. This effect, which is independent from the LDL-C reduction, could be also relevant since endothelin-1 is a well-known proatherogenic factor [33]. Finally, phytosterols seem to exert an anti-inflammatory action and inhibit insulin signaling [34]. Moreover, several preclinical findings and some preliminary clinical trials suggest that plant supplementation could improve the laboratory pattern of patients with non-alcoholic fatty liver disease (NAFLD) [35]. All these effects support the potential use of dietary supplementation with phytosterol in hyperlipidemic individuals as an adjunct to a healthy diet [36].

In the DESCO study, we tested a new ready-to-drink dietary supplement containing 2.5 g of plant sterols to evaluate its LDL-C-lowering effect, as well as the interaction between plant sterols and the quality of the diet on the lipid profile. At 3-week follow-up, supplementation with phytosterols, as expected, was associated with significant improvement in plasma TC, LDL-C, and Apo B-100 compared to baseline and as an adjunct to their standard diet. TC and LDL-C reduction were also significantly reduced when compared to placebo. The dietary supplement was well tolerated and compliance with the treatment was high. The LDL-C reduction we observed was higher than that obtained with plant sterol-enriched foods such as bread, biscuits, and cereals, but lower than that registered with plant sterol-enriched foods such as butter, margarine, and spreads [37]. This is potentially relevant considering that a standard Mediterranean diet contains a large amount of bread and cereals but a low content of butter, margarine, and spreads. Furthermore, our results are in line with those from a very recent meta-analysis of randomized clinical trials showing the cholesterol-lowering efficacy of plant sterol supplementation in capsules or tablets [38]. The intake of plant sterols as dietary supplements, rather than in enriched foods, has the advantages of greater feasibility of quantification and the ability to ensure the necessary doses, as well as easy acceptability and insertion in the context of diet therapy.

Interestingly, in our study, greater adherence to the Mediterranean diet significantly correlated with a more relevant clinical improvement in LDL-C (−9.2 ± 11.8%) and TC (−12.3 ± 8.6%), thereby suggesting a potential synergy between phytosterol and other active components of the Mediterranean diet. A possible explanation of this observation might be the larger quantity of polyphenols usually found in the Mediterranean diet [39,40]. Since these compounds have been shown to exert a slight inhibition of cholesterol synthesis due to the inhibition of HMGCoA reductase, their effect might theoretically potentiate the hypocholesterolemic effects of phytosterols acting on cholesterol absorption from the gut [39,40] Our preliminary observation of a potential additive effect of plant sterol efficacy from good adherence to the Mediterranean diet is also useful considering that the Mediterranean diet per se has proven to be an effective means to prevent ASCVD [11,41]. In fact, compared to different healthy dietary approaches, it has been estimated that the Mediterranean diet is associated with a significant reduction in the risk of cardiovascular mortality (odds ratio 0.55, 95% CI 0.39 to 0.78), stroke (odds ratio 0.65, 95% CI 0.46 to 0.93), and non-fatal myocardial infarction (odds ratio 0.48, 95% CI 0.36 to 0.65) [42]. Therefore, increasing the cholesterol-lowering ability of a healthy diet could further improve its preventive potential. Of course, this has to be yet demonstrated. Nonetheless, the hypothesis is reliable, considering the strong relationship existing between LDL-C plasma level and cardiovascular disease risk [43].

Despite its many strengths, we acknowledge that our study has some limitations. For instance, the washout period was short, so it could be argued that our outcome was affected by a carry-over effect. On the other hand, it must be considered that phytosterols, as previously mentioned, mediate their blood cholesterol-lowering effect via selectively inhibiting the absorption of cholesterol by the small intestine, without enzymatically interfering with its synthesis in the liver. For this reason, a carry-over effect is unlikely, especially because the active treatment period was short. Of course, the short duration of the supplementation could have partially reduced the observed treatment-dependent lipid-lowering effect. However, our purpose was to evaluate whether the tested treatment was able to exert a significant effect on LDL-C in the short term, i.e., the three weeks that the European regulation indicates is the useful time for obtaining a cholesterol-lowering effect. Crossover after washout and the subsequent re-evaluation of study endpoints strengthened the trial.

Of course, it is critically important that further longer-term clinical studies also investigate biomarkers for intestinal cholesterol absorption to ensure a more precise interpretation of the results. In effect, it is well known that there is a great interindividual variability in response to plant sterol supplementation [44]. Moreover, a deeper investigation of the interaction between the Mediterranean diet, its main bioactive components, and plant sterol supplementation needs to be implemented. This will remain the aim of further research by our group. Since we estimated the adherence to the Mediterranean diet with a questionnaire validated on the Italian population only, the eventual test of our experimental hypothesis on non-Italian populations would require the use of a different tool. On the other hand, the DESCO study showed that the once-a-day ready-to-drink dietary supplement we tested is able to quickly and significantly exert a lipid-lowering effect. Moreover, the product is characterized by convenience in terms of both storage (it can be kept at room temperature) and consumption (it is ready to use).

Overall, once our data are confirmed on larger trials, the tested product could be an effective and safe treatment option to improve cholesterolemia in the population and, consequently, a possible ethical business opportunity for food companies. 

## 5. Conclusions

Findings from the DESCO study show that the new ready-to-drink formulation of phytosterols we tested is well tolerated and quickly effective in reducing plasma cholesterol in moderately hypercholesterolemic individuals. In the study population, the effect was improved among individuals with a better adherence to the Mediterranean diet.

## Figures and Tables

**Table 1:** Quantitative composition of the active treatment tested in the clinical study.

Ingredients	Quantity per Stick (15 mL)
**Active Ingredients**	
Non-GMO Natural Mixed Phytosterols	2.50 g
Vitamin B1 (Thiamine hydrochloride)	0.42 mg
Vitamin B1 content	0.33 mg
**Main excipients**	
Fructose	1.5 g
Natural flavors	0.066 g
Sodium carboxymethyl cellulose	0.00375 g
Demineralized water	e.q. to 15 mL

e.q. = enough quantity.

**Table 2:** Anthropometric, hemodynamic, and blood chemistry parameters before and after the run-in period, expressed as mean ± standard deviation (SD).

Parameters	Pre-Run-in	Post-Run-in
Age (years)	51.5 ± 13.7	51.6 ± 13.6
Weight (kg)	65.4 ± 10.5	65.2 ± 10.1
Height (m)	1.65 ± 0.08	1.65 ± 0.08
Waist Circumference (cm)	86.3 ± 11.6	86.0 ± 11.4
Body Mass Index (kg/m^2^)	23.8 ± 3.2	23.7 ± 3.1
Index of Central Obesity	0.52 ± 0.06	0.52 ± 0.06
Lipid Accumulation Product	39.1 ± 39.5	39.2 ± 32.0
Visceral Adiposity Index	3.40 ± 2.44	3.58 ± 2.19
SBP (mmHg)	129.3 ± 9.7	128.9 ± 12.7
DBP (mmHg)	71.4 ± 7.0	71.3 ± 7.7
Total Cholesterol (mg/dL)	240.9 ± 22.8	237.1 ± 22.8
Triglycerides (mg/dL)	126.4 ± 87.0	124.6 ± 69.1
HDL-Cholesterol (mg/dL)	63.2 ± 15.3	63.6 ± 14.8
LDL-Cholesterol (mg/dL)	151.1 ± 20.8	148.6 ± 21.5
Non-HDL-Cholesterol (mg/dL)	177.7 ± 26.5	173.5 ± 25.3
VLDL-Cholesterol (mg/dL)	25.3 ± 17.4	23.1 ± 10.2
Apolipoprotein B-100 (mg/dL)	122.9 ± 18.0	119.8 ± 17.0

DBP = Diastolic blood pressure; HDL = High-density lipoprotein; LDL = Low-density lipoprotein; SBP = Systolic blood pressure; VLDL = Very-low-density lipoprotein.

**Table 3:** Anthropometric and hemodynamic parameter changes from the baseline to the end of the clinical trial, expressed as mean ± SD.

Parameters	Placebo (*n.* 50)		Phytosterols (*n.* 49)	
	Baseline	Week 3	Baseline	Week 3
Weight (Kg)	64.9 ± 10.2	64.8 ± 10.2	65 ± 10.1	64.6 ± 10
Body Mass Index (Kg/m^2^)	23.7 ± 3.1	23.6 ± 3	23.7 ± 3	23.6 ± 3
Waist Circumference (cm)	86 ± 11.6	85.8 ± 11.6	85.8 ± 11.3	85.7 ± 11.3
Index of Central Obesity	0.5 ± 0.1	0.5 ± 0.1	0.5 ± 0.1	0.5 ± 0.1
SBP (mmHg)	129.8 ± 12.8	130.1 ± 11.9	127.6 ± 15.2	128 ± 10.6
DBP (mmHg)	72.1 ± 8.8	70.7 ± 8.2	70.4 ± 7.4	69.2 ± 9.7
Lipid Accumulation Product	38.6 ± 26.9	36.5 ± 25.8	37.3 ± 26.7	37.1 ± 29.3
Visceral Adiposity Index	3.6 ± 1.9	3.5 ± 2	3.6 ± 2.3	3.4 ± 2.2
aMED score	6.0 ± 0.9	6.2 ± 1.1	6.1 ± 0.8	6.4 ± 1.2

aMED = Alternate Mediterranean; DBP = Diastolic blood pressure; SBP = Systolic blood pressure.

**Table 4:** Blood chemistry parameters from the baseline to the end of the clinical trial, expressed as mean ± SD.

Parameters	Placebo (*n.* 50)			Phytosterols (*n.* 49)		
	Baseline	Week 3	Mean Change from Baseline	Baseline	Week 3	Mean Change from Baseline
Total Cholesterol (mg/dL)	236.1 ± 23.5	233.9 ± 21	−2.2 ± 4.0	234.4 ± 22.2	224.6 ± 24.6 *	−11.8 ± 4.0 ^§^
LDL-C (mg/dL)	146.9 ± 22.2	146.6 ± 21.1	−0.3 ± 5.5	146.4 ± 21.3	138.6 ± 23.2 *	−7.8 ± 7.7 ^§^
HDL-C (mg/dL)	63.9 ± 14.6	63.4 ± 14	−0.4 ± 3.3	63.7 ± 14.7	61.7 ± 13	−2.1 ± 3.4
Non-HDL-C (mg/dL)	172.2 ± 26.5	170.3 ± 25.3	−1.8 ± 5.5	170.7 ± 22.4	166.2 ± 25.3	−4.5 ± 5.2
Triglycerides (mg/dL)	126.1 ± 60.1	119.5 ± 56	−4.6 ± 8.4	121.3 ± 58	121.6 ± 68	0.3 ± 8.7
VLDL-C (mg/dL)	25.2 ± 12	23.9 ± 11.2	−1.3 ± 2.3	23.9 ± 11.5	24.1 ± 13.8	0.2 ± 2.6
Apolipoprotein B-100 (mg/dL)	118.6 ± 17	117.2 ± 7.2	−0.1 ± 4.4	118.0 ± 15.9	114.3 ± 16.8 *	−3.7 ± 4.1

* *p* < 0.05 versus baseline; ^§^ *p* < 0.05 versus placebo. HDL-C = High-density lipoprotein cholesterol; LDL-C = Low-density lipoprotein cholesterol; VLDL-C = Very-low-density lipoprotein cholesterol.

**Table 5:** Multiple regression model investigating the main predictors of LDL-C reduction in the trial.

Model	Predictors	Non Standardized Coefficient		Standardized Coefficient	t	*p*	95% Confidence Interval	
		B	Standard Error	Beta			Lower Limit	Upper Limit
1	Active treatment	2.149	0.124	0.801	7.221	0.008	1.324	2.576
2	Active treatment	2.177	0.121	0.828	7.326	0.009	1.346	2.619
	Pre-treatment LDL-C	1.301	0.071	0.377	4.123	0.002	1.151	1.426
3	Active treatment	2.201	0.128	0.854	7.422	0.011	1.354	2.744
	Pre-treatment LDL-C	1.397	0.088	0.519	4.466	0.005	1.223	1.568
	aMED score	1.221	0.101	0.238	2.911	0.039	1.015	1.481

aMED = Alternate Mediterranean; LDL-C = Low-density lipoprotein cholesterol.

## Data Availability

Data supporting study’s findings are available from the Corresponding Author with the permission of the University of Bologna.
